# Transcriptome profiling reveals histone deacetylase 1 gene overexpression improves flavonoid, isoflavonoid, and phenylpropanoid metabolism in *Arachis hypogaea* hairy roots

**DOI:** 10.7717/peerj.10976

**Published:** 2021-03-16

**Authors:** Liangchen Su, Shuai Liu, Xing Liu, Baihong Zhang, Meijuan Li, Lidan Zeng, Ling Li

**Affiliations:** 1 Guangdong Provincial Key Laboratory of Biotechnology for Plant Development, South China Normal University, Guangzhou, Guangdong, China; 2 Department of Bioengineering, Zunyi Medical University, Zhuhai, Guangdong, China

**Keywords:** Histone deacetylase, Cell metabolism, Peanut, Growth of hairy roots, RNA-seq

## Abstract

**Background:**

The peanut (*Arachis hypogaea*) is a crop plant of high economic importance, but the epigenetic regulation of its root growth and development has not received sufficient attention. Research on *Arabidopsis thaliana* has shown that histone deacetylases (HDACs) are involved in cell growth, cell differentiation, and stress response. Few studies have focused on the role of HDACs in the root development of other plants, particularly crop plants. In earlier studies, we found large accumulations of *A. hypogaea* histone deacetylase 1 (*AhHDA1*) mRNA in peanut roots. However, we did not explore the role of AhHDA1 in peanut root development.

**Methods:**

In this paper, we investigated the role of the peanut *AhHDA1* gene and focused on the effect of altered *AhHDA1* expression in hairy roots at both the phenotypic and transcriptional levels. We analyzed the transformation of *A. hypogaea* hairy roots using Agrobacterium rhizogenes and RNA sequencing to identify differentially expressed genes that were assigned to specific metabolic pathways. Transgenic hairy roots were used as experimental material to analyze the downstream genes expression and histone acetylation levels. To thoroughly understand *AhHDA1* function, we also simultaneously screened the *AhHDA1*-interacting proteins using a yeast two-hybrid system.

**Results:**

*AhHDA1*-overexpressing hairy roots were growth-retarded after 20 d in vitro cultivation, and they had a greater accumulation of superoxide anions and hydrogen peroxide than the control and RNAi groups. *AhHDA1* overexpression in hairy roots accelerated flux through various secondary synthetic metabolic pathways, as well as inhibited the primary metabolism process. *AhHDA1* overexpression also caused a significant upregulation of genes encoding the critical enzyme chalcone synthase (*Araip.B8TJ0*, CHS) in the flavonoid biosynthesis pathway, hydroxyisoflavanone synthase (*Araip.0P3RJ*) in the isoflavonoid biosynthesis pathway, and caffeoyl-CoA O-methyltransferase (*Aradu.M62BY, CCoAOMT*) in the phenylpropanoid biosynthesis pathway. In contrast, ferredoxin 1 (*Araip.327XS*), the polypeptide of the oxygen-evolving complex of photosystem II (*Araip.N6ZTJ*), and ribulose bisphosphate carboxylase (*Aradu.5IY98*) in the photosynthetic pathway were significantly downregulated by *AhHDA1* overexpression. The expression levels of these genes had a positive correlation with histone acetylation levels.

**Conclusion:**

Our results revealed that the relationship between altered gene metabolism activities and *AhHDA1* overexpression was mainly reflected in flavonoid, isoflavonoid, and phenylpropanoid metabolism. *AhHDA1* overexpression retarded the growth of transgenic hairy roots and may be associated with cell metabolism status. Future studies should focus on the function of AhHDA1-interacting proteins and their effect on root development.

## Introduction

During the various stages of development and abiotic stress response, plant cells regulate the expression of many genes (Gan, Huang & Ito, 2013; Lee et al., 2016). Chromatin structure, which plays a crucial role in transcriptional regulation, is controlled by multiprotein complexes that recognize and instigate the biochemical modifications of histone proteins (Chen & Wu, 2010; Ganai et al., 2016). In these multiprotein complexes, histone acetyltransferase proteins (HATs) and histone deacetylases (HDACs) are the core catalyzing enzymes, and chromatin structure is maintained in a state of dynamic equilibrium (Mikkelsen et al., 2007). Therefore, HATs activate gene transcription through the acetylation of lysine residues in the N-termini of histones H3 and H4, which change the associated chromatin from a compacted to looser state. In contrast, HDACs function as antagonists to HATs by removing acetyl groups from histone acetylation sites, which causes transcriptional repression and gene silencing (Xing & Poirier, 2012; Ma et al., 2013). A dynamic balance between HAT and HDAC activity is important for the epigenetic regulation of the genome. Recent evidence has revealed that histone acetylation regulation plays a pivotal role in plant responses to salinity, drought, and other stresses (Kim et al., 2015).

HDACs have been isolated, identified, and are well conserved across species, including *Arabidopsis*, rice, maize, grapevine, and peanut (*Arachis hypogaea*) (Pandey et al., 2002; Aquea, Timmermann & Arce-Johnson, 2010; Ding et al., 2012; Su et al., 2015; Yang et al., 2016). Most HDACs in a given plant species belong to the RPD3/HDA1-like category (Ma et al., 2013). It has been reported that HDACs contribute to plant growth, development, stress responses, gene silencing, and other cellular processes such as cell death and the cell cycle. HDA6, HDA9, and HDA19 are the most studied HDACs. HDA6 participates in various plant hormone responses, including jasmonic acid (JA)- and abscisic acid (ABA)-mediated plant defense responses, and auxin responses related to transgene silencing. Recently, the *hda6* mutant was found to have a brassinosteroid (BR)-repressed phenotype in the dark and less sensitivity to BR biosynthesis inhibitors (Murfett et al., 2001; Devoto et al., 2002; Tanaka, Kikuchi & Kamada, 2008; Luo et al., 2012; Hao et al., 2016). HDA19 is involved in JA and ethylene signaling during the stress-response process and, together with HDA6, contributes to the inhibition of embryonic properties during germination (Zhou et al., 2005; Tanaka, Kikuchi & Kamada, 2008). HDA9 negatively regulates seed germination and seedling development (Van Zanten et al., 2014). Other HDACs have been reported to play a role in plant growth and development. For instance, HDA5 forms a protein complex with MULTICOP SUPPRESSOR OF IRA1 4/FVE (MSI4/FVE), FLOWERING LOCUS D (FLD), and HDA6 that is involved in flowering time regulation (Luo et al., 2015). Studies conducted on *Arabidopsis* (Cigliano et al., 2013; Liu et al., 2013b; Liu et al., 2013a) have shown that overexpression, as well as HDA7 silencing, can cause growth delays during post-germination and later developmental stages, while HDA7 downregulation can decrease silique fertility. In the *Arabidopsis* root epidermis, HDA18 regulates kinase genes involved in positional information signaling during cellular patterning.

Environmental adaptability, plant growth, and development are the major factors affecting crop breeding. Histone acetylation modification through epigenetic regulation is a new method expected to meet breeding scientists’ expectations. Researchers have found elevated *OsglHAT1* expression, grain-weight quantitative trait locus, and enhanced grain weight and yield in rice treated with increased acetylation levels of histone H4 (Song et al., 2015). Others have reported that the peanut allergy gene *Ara h 3* increased during histone H3 acetylation and decreased during histone H3K9 dimethylation of embryos at early maturation stages (Fu et al., 2010), and histone deacetylation modification repressed the peanut seed storage protein gene *Ara h 2.02* during germination (Yang et al., 2015). The results of these studies indicate that crop production and cell growth can be regulated by controlling histone acetylation levels, which are dynamically regulated by HATs and HDACs.

In our previous studies, *A. hypogaea* histone deacetylase 1 (*AhHDA1*), a histone deacetylase gene belonging to the HDAC family, was isolated and significantly upregulated under water deficit conditions. *AhHDA1* displayed higher expression in roots than in other organs (Su et al., 2015). However, the role that *AhHDA1* plays in peanut roots has not been explored. In this paper, we aimed to determine the function of *AhHDA1* using transgenic hairy roots. Modifications in *AhHDA1* expression, particularly overexpression, had a significant morphological effect on hairy root cells. Furthermore, transcriptome sequencing found that *AhHDA1* regulates the biosynthesis of various carbon-metabolism-related biological molecules. Our results provide insight into the role of *AhHDA1* in peanut hairy roots, lay a foundation for a more comprehensive understanding of histone deacetylase function in the peanut, and can be used for the breeding of potential new varieties.

## Materials & Methods

### Plant materials and growth conditions

Peanuts (*Arachis hypogaea* L. cv Yueyou 7) were sown in a 1:1:1 potting mixture of soil, vermiculite, and perlite (Fang et al., 2007). Plants were grown in an illuminated incubator with 16 h of light (200 µmol m^−2^ s^−1^, 26 °C) followed by eight hours of darkness (22 °C) (Su et al., 2015).

### Agrobacterium strain and binary vectors

We used the cucumopine-type *A. rhizogenes* strain K599 to induce peanut transgenic hairy roots. Using the cauliflower mosaic virus (CaMV) 35S promoter, the AhHDA1 cassette was released from *pCanG-AhHDA1*, which had been previously constructed by the pCanG-vector to form the overexpressing *AhHDA1* recombinant plasmid. The *AhHDA1* interference recombinant plasmid also used pCanG for its constructed backbone. We chose the 542 bp sense sequence, and designed the forward (*AhHDA1-sense-F*: 5′-ccgctcgagGACGTTGGTGTTGGCTCAGG-3′) and reverse (*AhHDA1-sense-R*: 5′-cccaagcttCTCTCCATGTCCTCTTCTGCC-3′) primer sequences. Antisense primer sequences were designed in forward (*AhHDA1-antisense-F*: 5′-CGAGCTCCTCTCCATGTCCTCTTCTGCC-3′) and reverse (*AhHDA1-antisense-R*: 5′-CTAGACTAGTGACGTTGGTGTTGGCTCAGG-3′). Together with the original plasmid *35S::eGFP*, the resulting binary constructs, *35S::AhHDA1-eGFP* and *35S::AhHDA1-RNAi*, were separately transformed into strain K599. The neomycin phosphotransferase gene (NPTII), controlled by the nopaline synthase (NOS) promoter located within the T-DNA, enabled positive transformants to be selected by kanamycin. Peanut hairy root induction resulted in transgenic hairy root formation (Liu et al., 2016b).

### Microscopic observation of hairy root tips

After the peanut hairy roots were cultured for 20 d and underwent *A. rhizogenes*-mediated transformation, we harvested 50 independent hairy root cell clones derived from different root points. Hairy root apical cells were photographed using an inverted microscope (Leica DMI3000 B, Wetzlar, Germany) and assessed using Digimizer 4.5 software. *A. thaliana* cell fluorescence was evaluated 1 d after infiltration using the LSM-800 confocal microscope (Zeiss, Oberkochen, Germany).

### Yeast two-hybrid analysis

After treating the samples with 30% PEG6000 (W/V) for five hours, we extracted the total RNA in the peanut roots, stems, and leaves to construct the yeast cDNA library. The cDNA fragments were ligated to pGADT7 plasmid and stored in yeast cells. Full-length *AhHDA1* cDNA was amplified using PCR and the following primer pairs: *AhHDA1-EcoRI-F*: 5′-TACGAATTCATGGGGATAGAAGAAGAGAG-3′, and *AhHDA1-SacI-R*: 5′-GAGCTCTCAGCAGCATCCATGTGG-3′. The PCR fragment was ligated into the vector pGBKT7 (Clontech, Mountain View, CA, USA) after cleavage by the restriction endonucleases *Eco* RI and *Sac* I (New England Biolabs, Ipswich, MA, USA). After all cloned fragments were transformed and screened using SD/Trp ^−^/Leu ^−^/His ^−^ selective medium, they were checked by PCR and sequenced. We performed the two-hybrid assays using the Matchmaker™ Gold two-hybrid system (Clontech) following the manufacturer’s instructions (Liu et al., 2016a).

### Measuring intracellular reactive oxygen species (ROS)

We used a superoxide anion radical chemiluminescent probe (Cat No. 075-05111, C_28_H_22_N_4_O_6_ , Wako, Osaka, Japan) and a highly selective fluorescent BES-H_2_O_2_-Ac H_2_O_2_ probe (Cat No. 028-17811, C_28_H_11_F_7_O_8_S, Wako) to measure superoxide anion content and H_2_O_2_ content, respectively, in addition to intracellular ROS levels (Kawase et al., 2004; Teranishi, 2007). After washing with phosphate-buffered saline (PBS) solution, we soaked the hairy roots in the PBS solution with the BES-H_2_O_2_-Ac fluorescence probe and the superoxide anion chemiluminescent probe for 20 min. The images were recorded using a laser scanning confocal microscope (Leica TCS SP8; Yamamoto et al., 2017). We then ground and homogenized the samples in phosphate buffer solution (pH 7.4) and collected the supernatant after centrifugation. We measured plate-reader-based luminescence in 96-well plates using a multimode reader (at 488 nm and 610 nm wavelengths, SpectraMax M5, Molecular Devices, San Jose, CA, USA) with the temperature set at 37 °C.

### RNAseq and data analysis

After 20 d of growth, we collected all transgenic hairy roots. *eGFP* expression in the transgenic hairy roots was detected using PCR and genomic DNA. The positive clones and hairy roots identified when testing for *AhHDA1* expression were chosen for RNA-seq. We used at least ten different positive hairy roots as independent biological replicates in each group for transcriptome analysis. The raw read *35S::eGFP*, *35S::AhHDA1-eGFP*, and *35S::AhHDA1-RNAi* groups were submitted to the NCBI database under Bioproject PRJNA395475 using the standard processing flow. The clean reads were sorted out by removing reads containing adapter, reads containing polyN, and low-quality reads from the raw data and aligned them to the peanut genome (http://peanutbase.org/home) using TopHat2 (version 2.0.3.12). The gene expression level was quantified using FPKM (Fragments Per Kilobase of transcript per Million mapped reads) method, which calculated the FPKM of each gene based on the gene’s length and read count. We used the negative binomial (NB) distribution which was simulated by edgeR software for differential expression analysis, and the false discovery rates was calculated by BH algorithm. Genes with false discovery rates ≤ 0.05 and with more than twice the difference between the control group and either the overexpressing *AhHDA1* or the *AhHDA1* interference group, were considered differentially expressed.

### Reverse transcription and real-time PCR

We carried out RNA extraction using the methods described by Su et al. (2015). Three biological RNA sample replicates for each time point and treatment were used for downstream applications, and the relative expression for each well was calculated (Muller et al., 2002). We normalized the *A. hypogaea* L expression data using the geometric mean (geomean) of the validated housekeeping gene. We specifically used the peanut *ACTIN* gene (GenBank accession no. GO339334) to amplify a fragment of 108 bp (Chi et al., 2012). The mean values and standard errors were calculated from the three biological replicates. The primers used for differentially expressed gene (DEG) expression verification are listed in Table S2.

### Chromatin immunoprecipitation assay (ChIP)

We collected 100 mg of hairy roots that had been cultivated for 20 d and cross-linked them using formaldehyde. The nuclear isolation method was performed as previously described by Saleh, Alvarez-Venegas & Avramova (2008). We used anti-H3ac ChIP, the specific antibody for H3ac (Cat. NO. 06-599, Millipore, Burlington, MA, USA), in this study. After sonication, protein complexes were precipitated using the acetyl-H3 antibody at 4 ° C overnight and captured with Magna ChIP™ Protein A+G Magnetic Beads (Cat. NO. 06-663, Millipore). The beads were washed and reverse cross-linked, and we performed DNA purification after the proteins were digested. The primers used for the real-time PCR experiments are listed in Table S2. Each sample was replicated at least three times.

## Results

### AhHDA1 belongs to the HDAC family and is located in the nucleus

**Figure 1:**
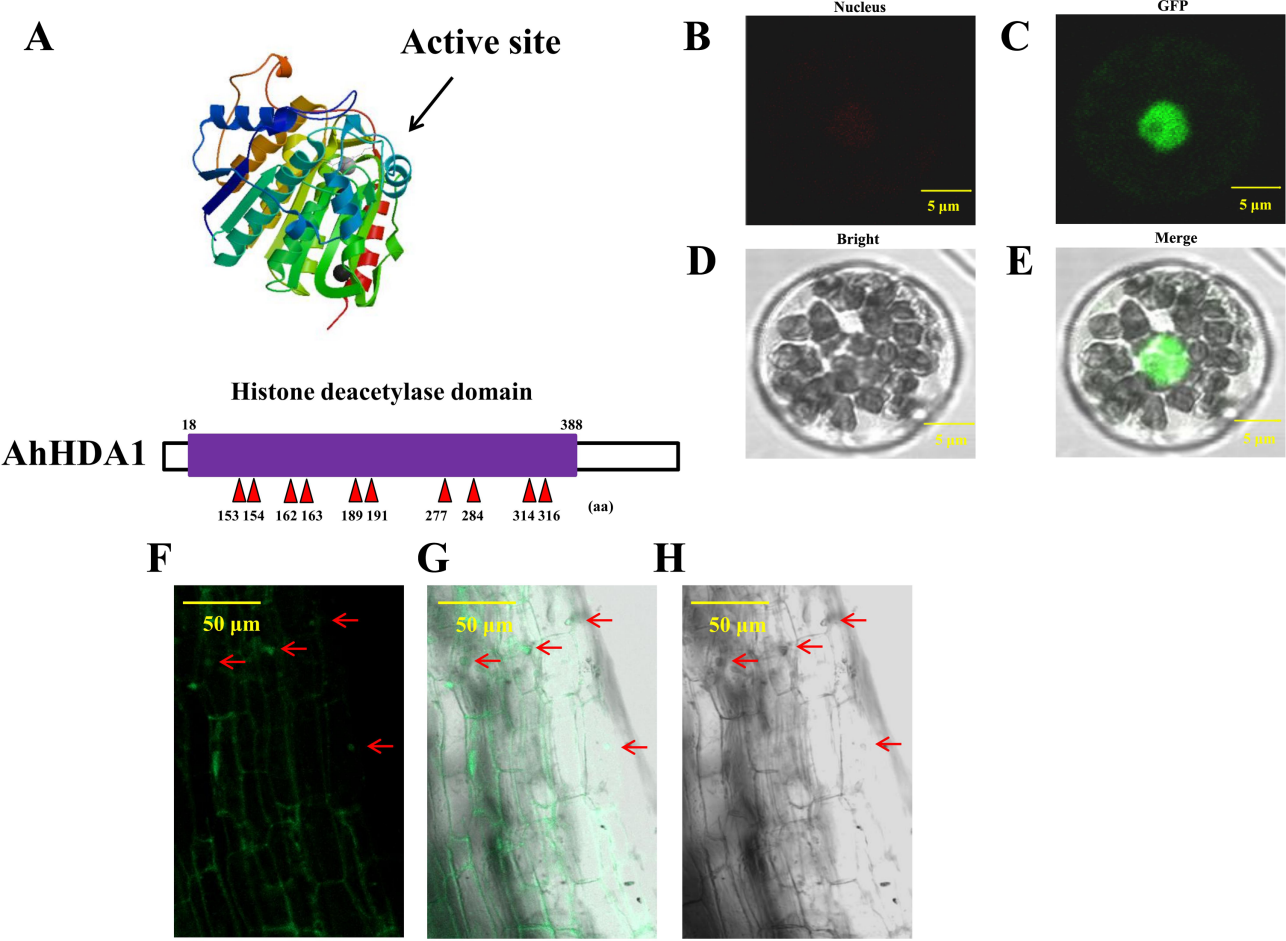
AhHDA1 protein posesses a typical histone deacetylase domain and locates in nucleus. (A) Histone deacetylase domain in AhHDA1. (B–E) AhHDA1 protein locates in nucleus in the transient transfection system of Arabidopsis thaliana. (F–H) Green fluorescence in *35S:: AhHDA1-eGFP* hairy root cells indicates that AhHDA1 is localized in the nucleus. The yellow bar stands for 50 µM.

We obtained *35S::AhHDA1* and *35S::AhHDA1-RNAi* transgenic hairy roots by transforming four-leaf peanut seedlings. We found a high degree of similarity between *AhHDA1* protein and other HDACs of the universal histone deacetylase domain from 18 to 388 amino acid sites. We predicted that 153, 154, 162, 163, 189, 191, 277, 284, 314, and 316 sites were active key positions where the enzyme plays an effective role in catalyzing histone deacetylation (Fig. 1A). *AhHDA1* protein was found in the nucleus, where it contributed to protoplast transformation and fluorescence localization in *35S::AhHDA1-eGF*-transformed hairy root cells (Figs. 1B–1H).

### *AhHDA1* overexpression retards growth in transgenic hairy roots

**Figure 2:**
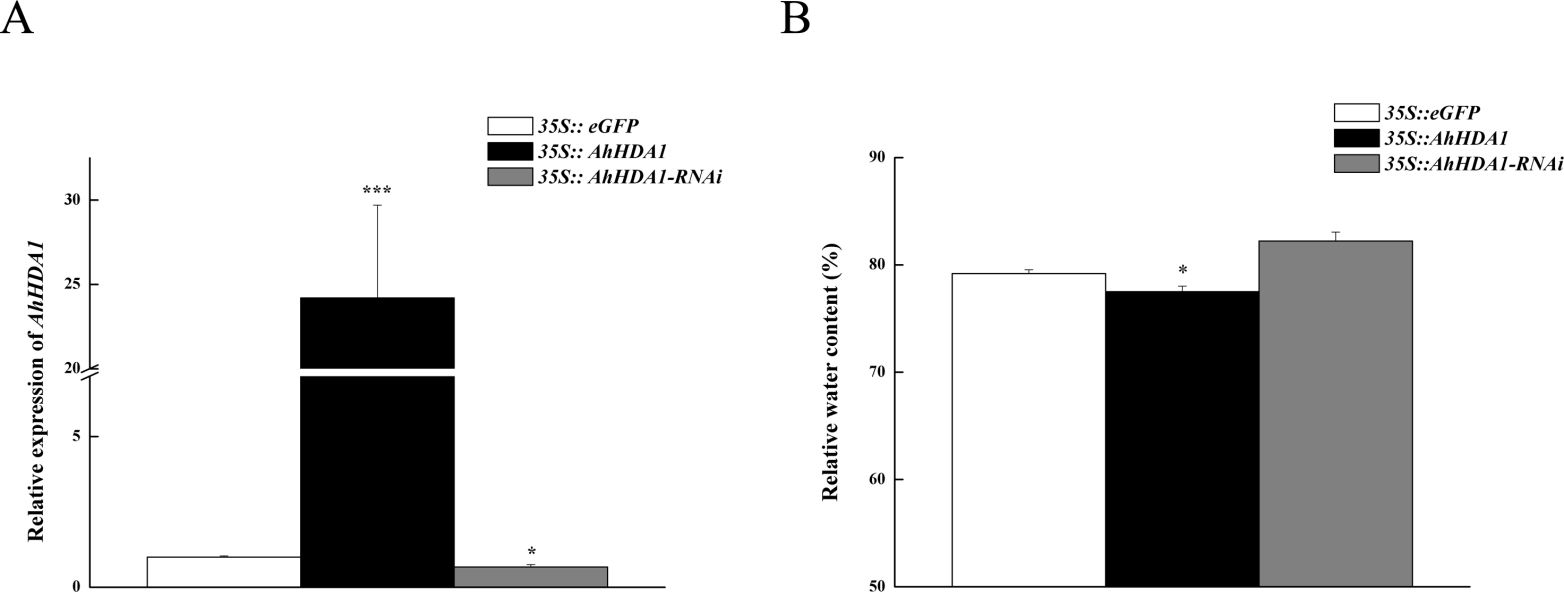
Overexpressed AhHDA1 shows a low relative water content. (A) Relative expression of AhHDA1 in different transgenic hairy roots. *35S:: eGFP* stands for the empty plasmid transformed; *35S:: AhHDA1* stands for *AhHDA1* overexpressing hairy roots; *35S:: AhHDA1*–*RNAi* stands for the RNA interfering hairy roots. (B) Relative water content in 20 d growth of different transgenic hairy roots.

**Figure 3:**
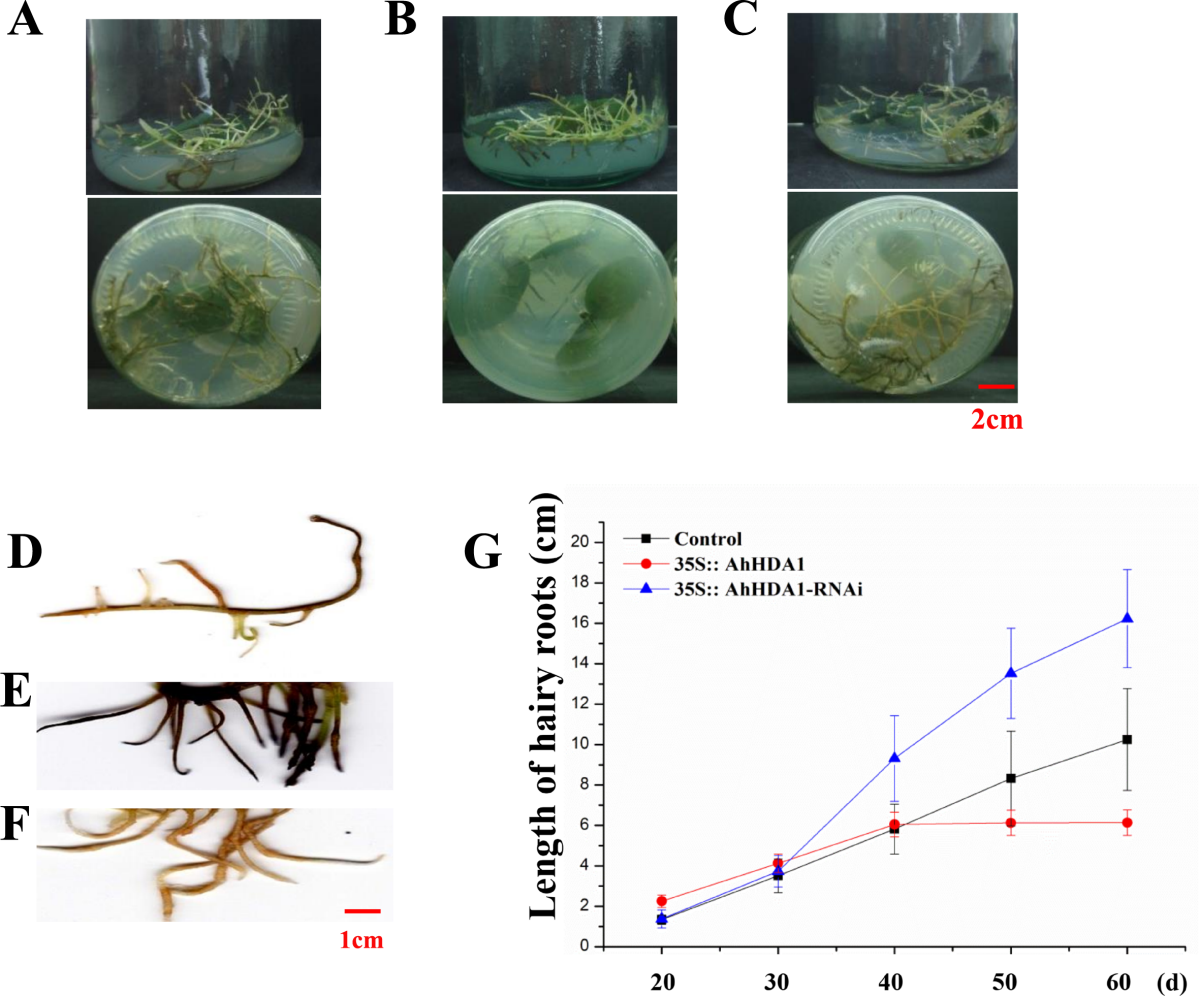
35S:: AhHDA1 transgenic hairy roots possess the characteristic of retarded growth. (A, D) Growth morphology of the *35S:: eGFP* transgenic hairy roots. (B, E) Growth morphology of the *35S:: AhHDA1* transgenic hairy roots. (C, F) Growth morphology of the *35S:: AhHDA1-RNAi* transgenic hairy roots. (G) Growth rate of transgenic hairy roots, the values are the means of at least 50 replicates with standard deviation.

To observe the influence of differentially expressed *AhHDA1* on cells, we used *AhHDA1* that had been growth-overexpressed for 20 d and hairy roots with *AhHDA1* interference. *AhHDA1*-overexpressed hairy roots had a relative expression 24.25 times that of the control group, while the relative expression of hairy roots with *AhHDA1* interference was 0.52 greater than that of the control group (Fig. 2A). The control group’s relative water content (RWC), overexpressed *AhHDA1* RWC, and hairy roots with *AhHDA1* interference RWC was 79.23%, 77.54%, and 82.36%, respectively. The RWC of the overexpressed *AhHDA1* hairy roots was significantly lower than the control group RWC (Fig. 2B). Meanwhile, the *35S::AhHDA1-eGFP* transgenic hairy roots became denser with darker coloration after 20 d in vitro cultivation (Fig. 3A and 3B). When the growth period was longer than 30 d, the *35S::AhHDA1-eGFP* transgenic hairy roots grew more slowly and eventually stopped growing entirely. In contrast, both the *35S::AhHDA1-RNAi* group and the control group maintained a relatively high growth rate, with the highest found in the *AhHDA1-RNAi* transgenic hairy roots (Fig. 3C).

**Figure 4:**
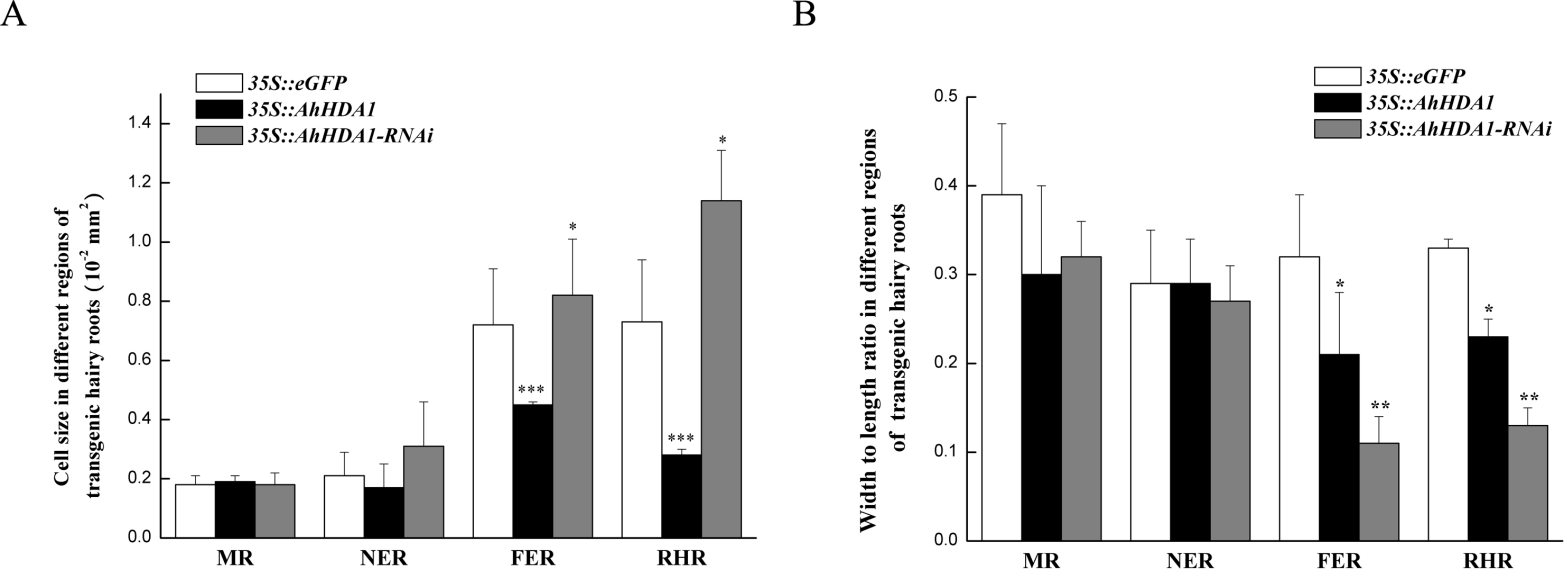
Morphologicobservation of different transgenic peanut hairy roots. MR: meristem region; NER: The near elongation region; FER: The far elongation region; RHR: Root hair region. (A) Cell size in different regions of transgenic hairy roots. (B) Width to length ratio in different regions of transgenic hairy roots. Each graph displays the means and SD of at least 50 replicates with standard deviation */* * /***, different from control as revealed by *t*-test, *P* < 0.05∕0.01∕0.001.

Under a light microscope, we observed the surface cells of the transgenic hairy roots that had been cultivated for 20 d. Although there was no significant difference in cell size across the different groups in the meristem and near elongation regions, the hairy root cells overexpressing *AhHDA1* were significantly smaller than the control cells and the *35S::AhHDA1-RNAi* cells in the root hair and far elongation regions (Fig. 4A). Furthermore, the cell width-to-length ratios in the *35S::AhHDA1* and *35S::AhHDA1-RNAi* transgenic hairy roots were smaller than in the control group, and these cells appeared to be a long and narrow form. Therefore, altered *AhHDA1* expression had an apparent effect on the morphology of cells near the root hair region (Fig. 4A and 4B).

**Figure 5:**
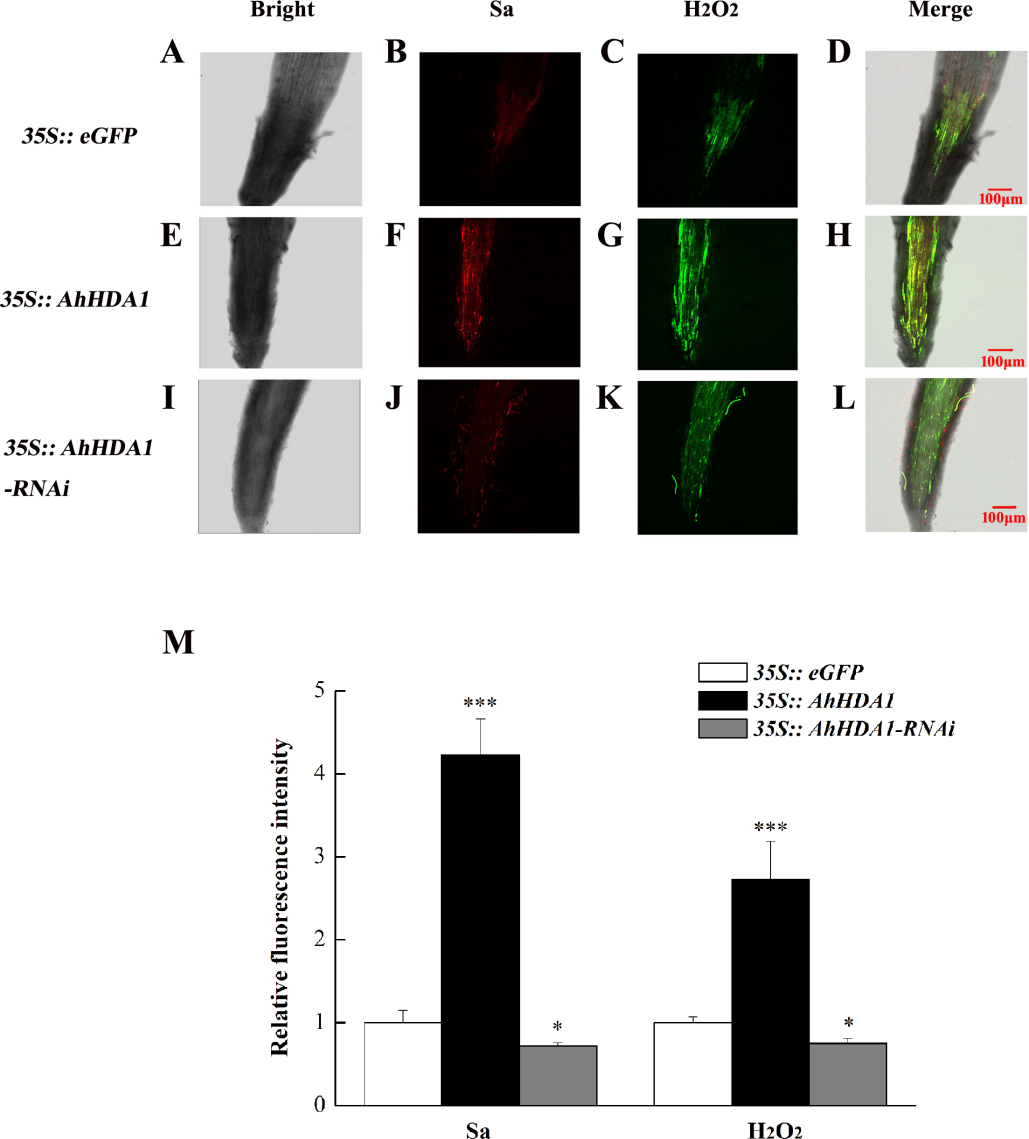
Reactive oxygen species have higher degree of accumulation in over-expressing *AhHDA1*. (A, E, I) Fluorescence images taking by laser scanning confocal microscope in different transgenic hairy roots in the bright light. (B, F, J) Fluorescence images taking by laser scanning confocal microscope in different transgenic hairy roots by 488 nm wavelengths. (C, G, K) Fluorescence images taking by laser scanning confocal microscope in different transgenic hairy roots by 610 nm wavelengths. (D, H, L) Merging fluorescence images taking by laser scanning confocal microscope in different transgenic hairy roots. (M) Relative fluorescence intensity of Sa and H_2_O_2_ in different transgenic hairy roots. Red fluorescence represents super oxygen anion (Sa) strength, Green fluorescence represents hydrogen peroxide (H_2_O_2_) content. The bar stands for 100 µM. Each graph displays the means and SD of at least 5 replicates with standard deviation. */***, different from control as revealed by *t*-test, *p* < 0.05∕0.001.

The intracellular ROS concentration reflects the extent to which cells are experiencing environmental stress. We used specific fluorescent probes to measure the relative concentration of superoxide anions and H_2_O_2_ in transgenic hairy roots. The superoxide anion concentration in the *AhHDA1* hairy roots that had been overexpressed for 15 d was 4.06 times greater than that of the blank control roots, while the *35S::AhHDA1-RNAi* group was 0.75 times greater than that of the control group. Similarly, the H_2_O_2_ concentration in overexpressed *AhHDA1* hairy roots was 2.81 times greater than the blank control levels, while the *35S::AhHDA1-RNAi* group was 0.77 times greater than that of the control group (Fig. 5).

### *AhHDA1* overexpression in hairy roots influences flavonoid, isoflavonoid, and phenylpropanoid metabolism expression

**Figure 6:**
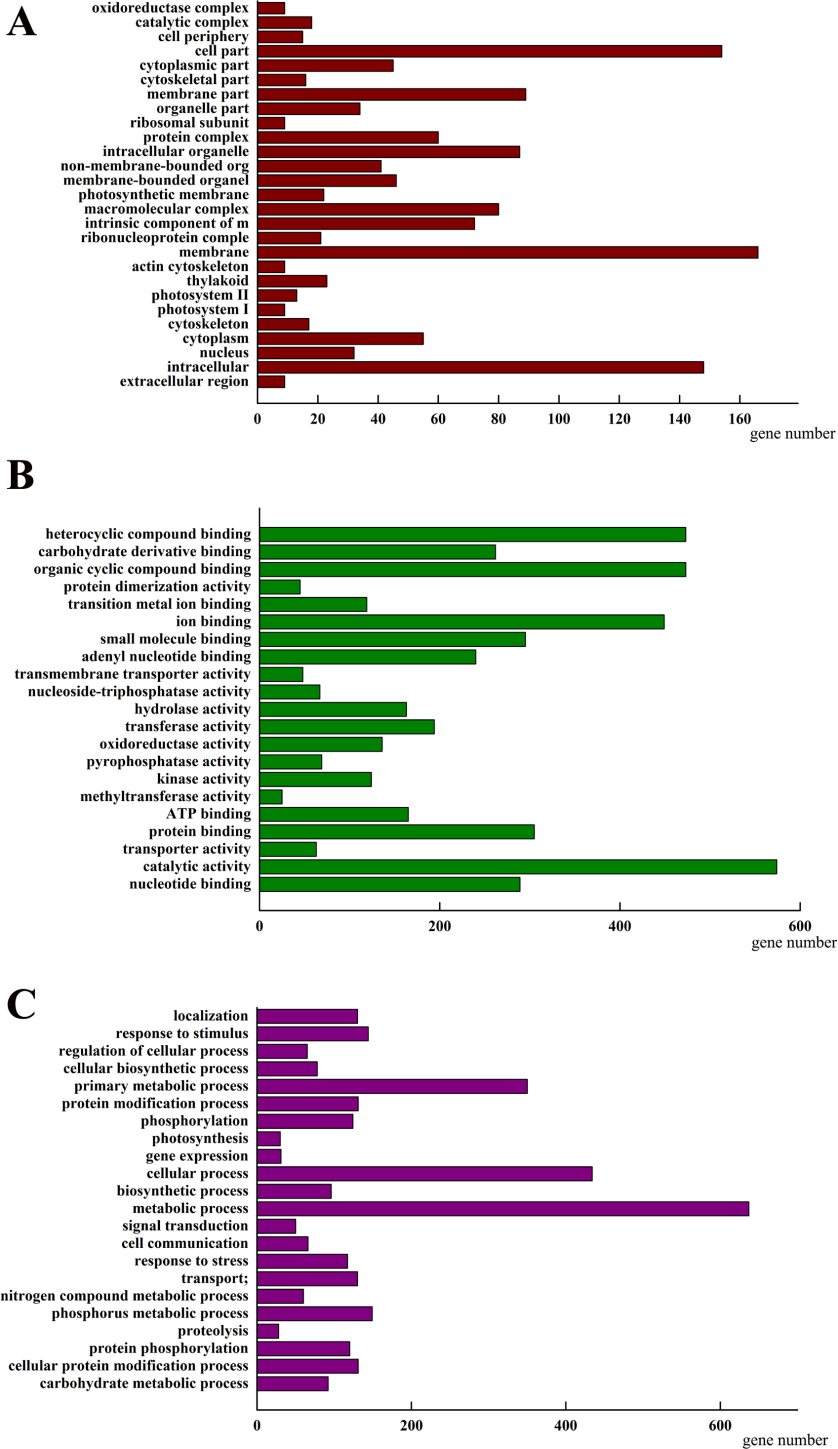
Classification of differentially expressed genes (DEGs) by GO (Genome annotation) enrichmentamong the control group, *35S:: AhHDA1* and *35S:: AhHDA1-RNAi* hairy roots. (A) DEGs by classified in cellular component (CC). (B) DEGs by classified in molecular function (MF). (C) DEGs by classified in biological process (BP).

To investigate how *AhHDA1* affects cell morphology in hairy roots, we obtained transcriptome data for both *35S::AhHDA1* and *35S::AhHDA1-RNAi* hairy roots (http://www.ncbi.nlm.nih.gov/Traces/study/?acc=SRP113568) to separately compare them to the control group and to identify the DEGs. Using the criteria of >2-fold difference in expression level and *p* <0.05, we found 2,157 DEGs when comparing the RPKM of different groups following GO enrichment classification (Fig. 6). Most DEGs influenced by *AhHDA1* were grouped under cell part, membrane, and intracellular in the cellular component category (166, 154, and 148 genes, respectively); binding (824 genes) and catalytic activity (574 genes) in the molecular function category; and metabolic process (637 genes) and oxidation–reduction process (179 genes) in the biological process category. It is worth noting that in the biological process category, a relatively large number of genes (50.49%) were related to metabolic processes, including primary metabolism, nitrogen compound metabolism, phosphorus metabolism, and carbohydrate metabolism.

**Figure 7:**
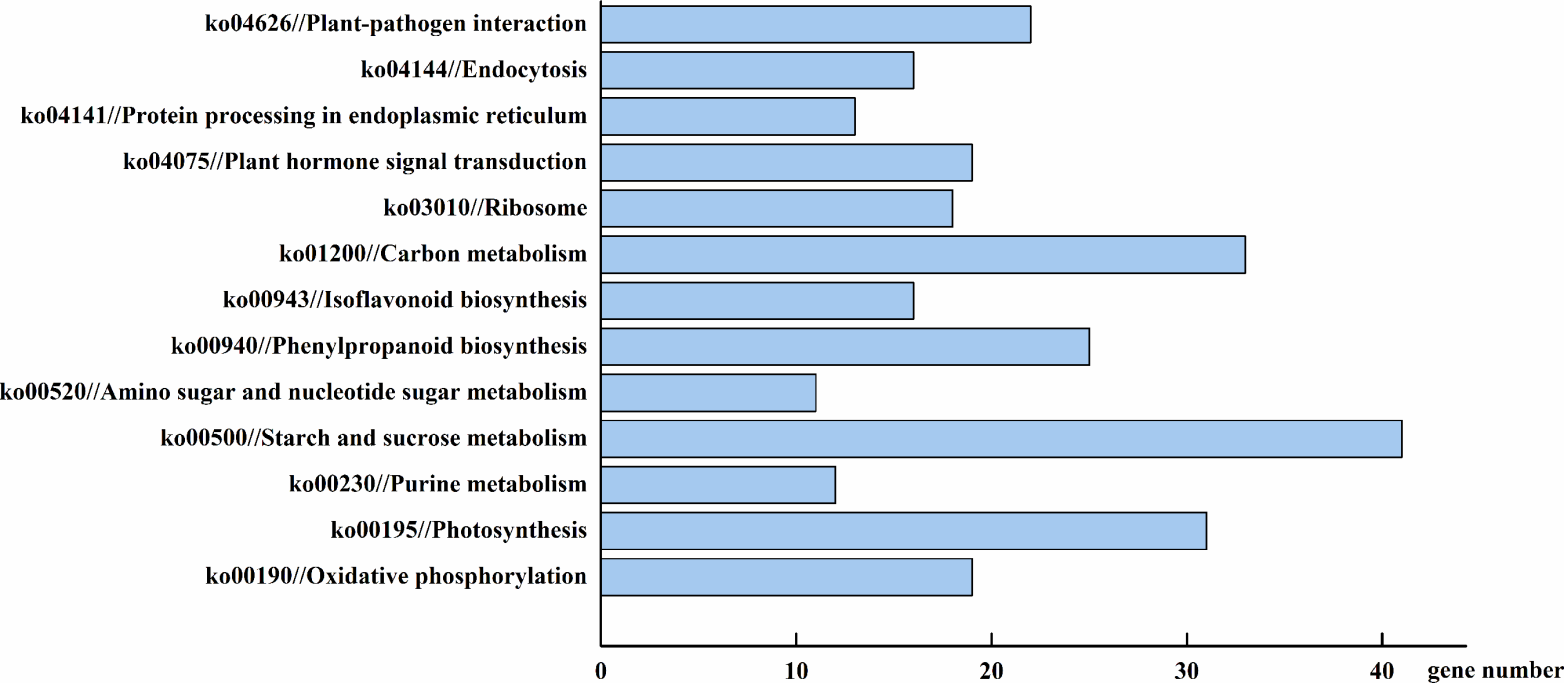
DEGs involved in major pathways AhHDA1 influenced by KEGG (Kyoto Encyclopedia of Genes and Genomes) analysis. Starch and sucrose metabolism, carbon metabolism and photosynthesis are the pathways with the most gene enrichment AhHDA1 influenced.

To more thoroughly understand the role of the identified DEGs, we examined their gene product pathways. Four hundred and sixty-eight of the total 2,157 DEGs could be assigned to a KEGG pathway (Fig. 7). The starch and sucrose metabolism (ko00500), carbon metabolism (ko01200), and photosynthesis (ko00195) pathways were the most gene-enriched pathways, with 41, 33, and 31 genes, respectively. Other pathways, including the phenylpropanoid biosynthesis (ko00940), oxidative phosphorylation (ko00190), plant hormone signal transduction (ko04075), and isoflavonoid biosynthesis (ko00943) pathways, were also modulated by AhHDA1’s effect on downstream gene expression. This result suggests that *AhHDA1* influences several biosynthetic and metabolic pathways. We screened 98 DEGs using a stricter filter criterion: the FPKM of either group had to be above 20 and the relative expression difference between either two groups had to be above 5. Among the screened DEGs, 50 were found to be related to the metabolism pathway, and 41 (not including photosynthesis-related DEGs) were found to be associated with substance synthesis and energy metabolism. We found that 31 of the 50 DEGs were upregulated in *AhHDA1* over-expressed hairy roots (Fig. S1). Meanwhile, 13 DEGs had an association with the photosynthesis process, and 11 of them were found downregulated in *AhHDA1* over-expressed hairy roots (Fig. S2). Sixteen DEGs were found to have an association with oxidative stress-related genes. Fourteen of the 16 DEGs were found upregulated in *AhHDA1* over-expressed hairy roots, and included four NAC domain proteins, three LEA proteins, two senescence-associated proteins, and two plant hormone-responsive proteins (Table S3). Our results imply that *AhHDA1* over-expression can change the cell metabolic and stress response abilities of peanut hairy roots.

**Figure 8:**
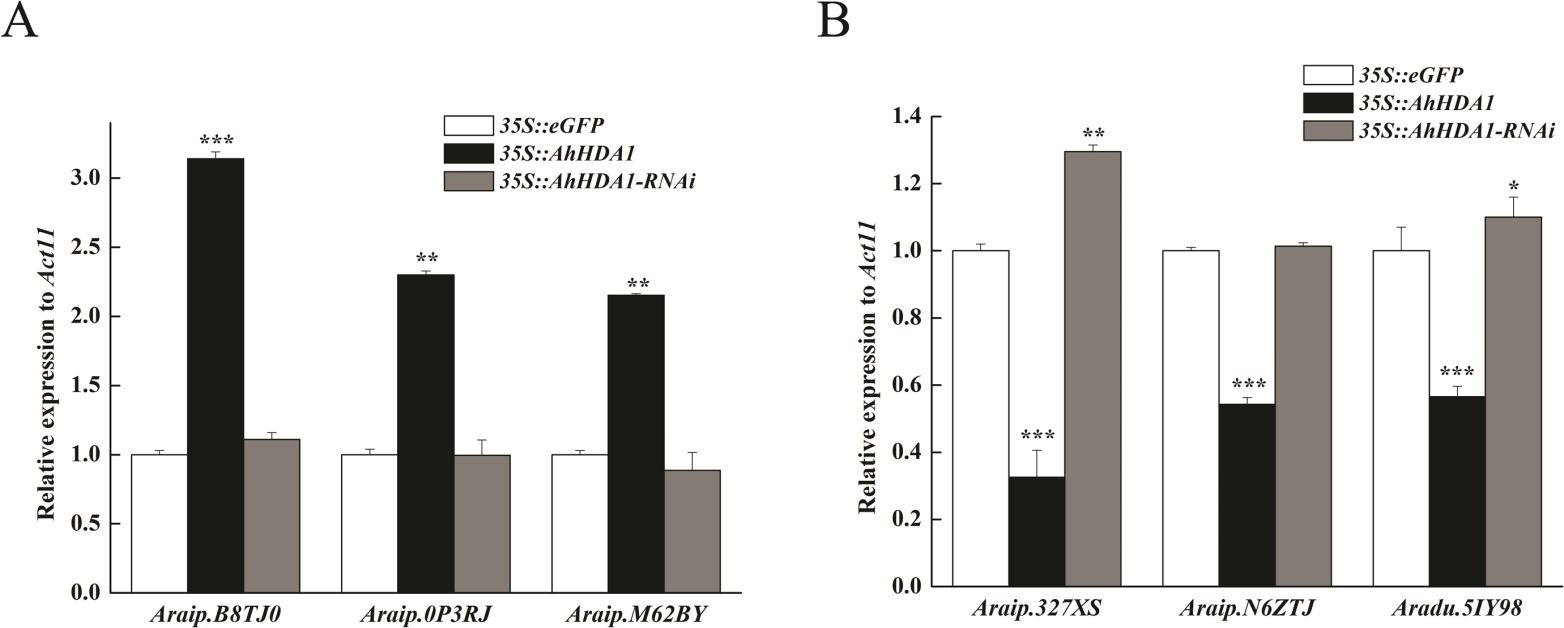
Transcript levels measured by real-time RT-qPCR in different transgenic hairy roots. (A) Transcriptional level of DEGs in flavonoid, isoflavonoid and phenylpropanoid biosynthesis pathways. (B) Transcriptional level of DEGs in photosynthetic pathway. *Act11* was used as an internal control. Results from experiments repeated three times, each with three qPCR measurements, indicating the mean ±  SEM, *n* = 3 replicates (6–8 plants were pooled for each measurement). Each graph displays the mean and SD of three independent experiments. */**/***, different from control as revealed by *t*-test, *P* < 0.05∕0.01∕0.001.

To verify the transcriptome results, we selected six DEGs thought to be important in carbon metabolism pathways and used real time-PCR (RT-PCR) to determine their expression levels. The DEGs were *Araip.B8TJ0* (chalcone synthase, CHS) in the flavonoid biosynthesis pathway; *Araip.0P3RJ* (hydroxyisoflavanone synthase) in the isoflavonoid biosynthesis pathway; *Aradu.M62BY* (caffeoyl-CoA O-methyltransferase, CCoAOMT) in the phenylpropanoid biosynthesis pathway; and *Araip.327XS* (ferredoxin 1), *Araip.N6ZTJ* (polypeptide of the oxygen-evolving complex of photosystem II), and *Aradu.5IY98* (ribulose bisphosphate carboxylase) in the photosynthetic pathway. We found that the transcriptional levels of *Araip.B8TJ0*, *Araip.0P3RJ*, and *Araip.M62BY* in the *35S::AhHDA1* transgenic group were significantly higher than in the control group and *35S::AhHDA1-* RNAi group. Additionally, the transcriptional levels of *Araip.327XS*, *Araip.N6ZTJ*, and *Aradu.5IY98* in the *35S::AhHDA1* transgenic group were significantly lower than in the other two groups (*p* <  0.001) (Fig. 8). This observation was consistent with the transcriptome data results.

*AhHDA1* overexpression enhanced the transcriptional level of phenylpropanoid, flavonoid, and flavonoid biosynthesis genes, all of which are involved in secondary metabolism. However, we found that the expression of photosynthetic-related genes was downregulated in over-expressed hairy roots. Higher expression levels of secondary metabolism genes and lower expression levels of primary metabolism genes may be related to slower growth rates in these transgenic plants.

### *AhHDA1* -interacting proteins and their predictive function

Since HDACs form a functional complex with other proteins, it is important to find *AhHDA1*-interacting proteins. Using the yeast two-hybrid technique, we identified 37 AhHDA1-interacting protein candidates: 19 transcription factors, 13 enzymes, and three molecular chaperones or their regulators and two other proteins. Some of the most interesting proteins that interacted with *AhHDA1* were Arabidopsis pseudo-response regulator-like (APRR2-like, 19.35%), Golden2-like (GLK1-like, 11.29%), basic Helix-Loop-Helix (bHLH77-like, 2.42%), NAC2-like transcription factors (2.42%), and MYB-like transcription factors (2.42%). Enzymes mostly function during cellular metabolic processes, and molecular chaperones or their regulators may contribute to nucleosome assembly. In addition to these examples, 18 proteins may be involved in cellular synthetic metabolic pathways. Nine proteins are related to stress responses and six proteins are related to photosynthesis and oxidative phosphorylation (Table 1).

**Table 1:** AhHDA1-interacting protein candidates identified by Y2H screening.

Seq.	Des.	ID.	Per.
1	Two-component response regulator-like APRR2-like	XM_014763821.1	19.35%
2	Transcription activator GLK1-like	XM_003543275.3	11.29%
3	TdnaJ protein homolog 2-like	XM_003529001.2	9.68%
4	TATP synthase delta subunit (atpD) gene	XM_021990240.1	5.65%
5	TABC1 family protein	XM_003588546.2	4.84%
6	Peroxisomal (S)-2-hydroxy-acid oxidase GLO1-like	XM_014778344.1	4.03%
7	Serine/threonine-protein kinase D6PK	XM_020375073.1	3.23%
8	F-box protein SKIP8	XM_012716710.1	2.42%
9	Transcription factor bHLH77-like	XM_002264933.4	2.42%
10	Protein CHUP1, chloroplastic-like	XM_006573213.2	2.42%
11	NAC2 transcription factor	EU755023.1	2.42%
12	BAG family molecular chaperone regulator 1-like	XM_006599571.2	2.42%
13	GTP-binding protein hflX	XM_003589279.2	2.42%
14	Inactive poly [ADP-ribose] polymerase RCD1	XM_011029811.1	2.42%
15	Peroxisomal biogenesis factor 6-like	XM_006589453.2	2.42%
16	12-oxophytodienoate reductase 3-like	XM_003542310.3	1.61%
17	Chloroplast translational elongation factor Tu (tufA)	AF234537.1	1.61%
18	Inactive poly [ADP-ribose] polymerase RCD1	XM_020347479.1	1.61%
19	Importin subunit alpha-1-like	XM_003536002.3	1.61%
20	Syntaxin (MTR_8g066340)	XM_003628703.2	1.61%
21	XJS-XC-01 ribulose-1,5-bisphosphate carboxylase small subunit	KF607110.1	1.61%
22	Activator inhibitor 1 RNA-binding protein-like	XM_003521018.3	1.61%
23	1-deoxy-D-xylulose 5-phosphate reductoisomerase (Dxr)	AY315651.1	1.61%
24	Small glutamine-rich tricopeptide containing protein (Sgt)	NM_001259129.2	1.61%
25	DNA-(apurinic or apyrimidinic site) lyase, chloroplastic-like	XR_001385255.1	1.61%
26	Cyclin-D-binding Myb-like transcription factor 1	XM_021111595.1	1.61%
27	Annexin	KM267643.1	0.81%
28	Cyclin-D-binding Myb-like transcription factor 1	XM_014654192.2	0.81%
29	Pre-mRNA-splicing factor SLU7-like protein	XM_013594443.1	0.81%
30	chloroplast-related	KX289923.1	0.81%
31	SNF1-related protein kinase catalytic subunit alpha KIN10-like	XM_014770944.1	0.81%
32	Protein CHUP1, chloroplastic-like	XM_006573213.2	0.81%
33	BAG family molecular chaperone regulator 3-like	XR_137352.3	0.81%
34	Protein kinase	XM_003625972.2	0.81%
35	Putative aryl-alcohol dehydrogenase	XM_013609480.1	0.81%
36	NADP-dependent glyceraldehyde-3-phosphate dehydrogenase-like	XM_003549502.3	0.81%

### Histone acetylation activity of *AhHDA1*-influenced downstream genes

**Figure 9:**
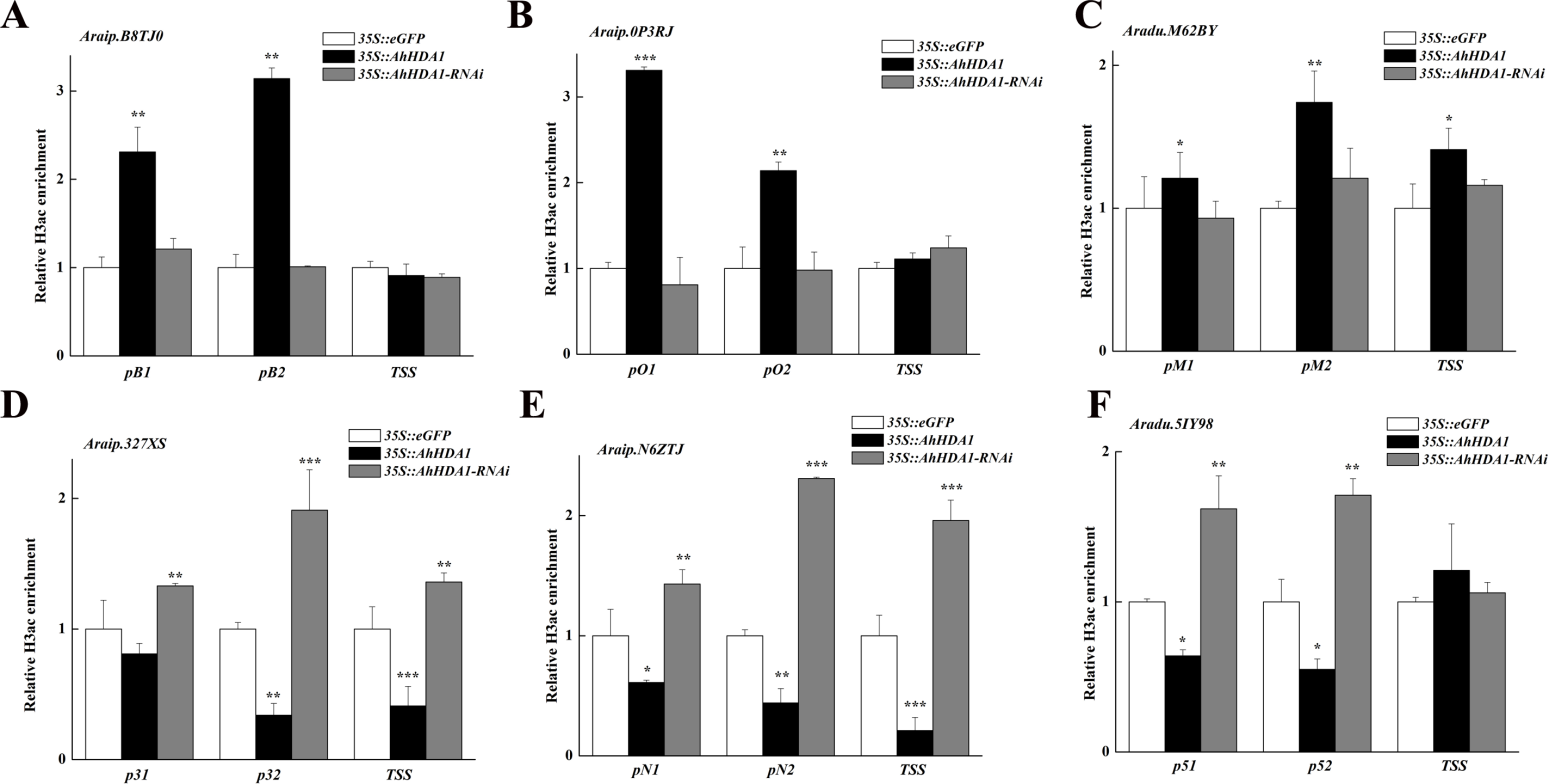
AhHDA1 affect the H3ac acetylation status at ABA metabolism related genes. (A) The graphs show increased H3ac levels in overexpressed *AhHDA1* hairy roots (black) and decreased H3ac levels in interference *AhHDA1* hairy roots (gray) at the *A raip.B8TJ0*, *Araip.0P3RJ* and *Araip.M62BY* locus, respectively. (B) The graphs show decreased H3ac levels in overexpressed *AhHDA1* hairy roots (black) and increased H3ac levels in interference *AhHDA1* hairy roots (gray) at the *Araip.327XS*, *Araip.N6ZTJ* and *Aradu.5IY98* locus, respectively. Values represent recovery expressed as relative enrichment of H3ac compared with *Actin11* levels. Results from experiments repeated three times, each with three qPCR measurements, indicating the mean ±  SEM, *n* = 3 replicates (6–8 plants were pooled for each measurement). Each graph displays the mean and SD of three independent experiments. */**/***, different from control as revealed by *t*-test, *P* < 0.05∕0.01∕0.001.

We analyzed the H3 acetylation antibody (H3ac) enrichment of promoters in these *AhHDA1*-influenced metabolism genes using ChIP-PCR. Increased *Araip.B8TJ0*, *Araip.0P3RJ*, and *Araip.M62BY* gene fold enrichment showed that the transcriptional activity of these genes was enhanced in overexpressed hairy roots. Decreased *Araip.327XS*, *Araip.N6ZTJ*, and *Aradu.5IY98* gene fold enrichment to H3ac showed reduced transcriptional activity in overexpressed hairy roots. The critical transcriptional activation regions of these genes were: *pB1* and *pB2* in *Araip.B8TJ0*, *pO1*, and *pO2* in *Araip.0P3RJ*, *pM2* in *Araip.M62BY*, *p32* and *TSS* in *Araip.327XS*, *pN2* and *TSS* in *Araip.N6ZTJ*, and *p51* and *p52* in *Aradu.5IY98* (Fig. 9). The histone acetylation of *Araip.B8TJ0*, *Araip.0P3RJ*, and *Araip.M62BY* genes in the *35S::AhHDA1-RNAi* group did not additionally decrease, while the histone acetylation of *Araip.327XS*, *Araip.N6ZTJ*, and *Aradu.5IY98* genes significantly increased.

## Discussion

The peanut (*Arachis hypogaea* L.), a source of protein with a seed oil content of about 45–56%, is one of the most economically profitable crops (Chen et al., 2016). The root is the main organ by which its crops absorb nutrients and water, making peanut root growth and developmental characteristics of considerable interest. We examined the role of epigenetics in peanut root growth and development because this field is important to crop plant breeding, yet largely unexplored.

### *AhHDA1* overexpression suppressed root development and changed apical cell morphology in peanut hairy roots

Earlier studies found that during root development, both HDA18 and HDA19 affected the root epidermal cellular pattern in *A. thaliana* (Liu et al., 2013a; Liu et al., 2013b; Chen et al., 2019). HDA6 affected the cellular pattern of *Arabidopsis* root epidermis by altering the histone acetylation status of some gene promoters (Li et al., 2015). In *AhHDA1* that was isolated in peanuts, the transcriptional level in the root was the highest (Su et al., 2015). In this paper, we investigated the role of *AhHDA1* in root development and found that *AhHDA1* overexpression retarded growth and decreased the RWC in hairy roots (Figs. 2 and 3). Under a light microscope, we observed significantly smaller root apical cell sizes in overexpressing plants than in other groups. Moreover, the width-to-length ratios in the hairy root cells of the overexpressing groups were smaller than that of the control group, and cells near the root hair region were long and narrow in shape (Fig. 4). The alterations in root morphology may also be related to root development.

### Transcriptome analysis indicated *AhHDA1* overexpression improves flavonoid, isoflavonoid, and phenylpropanoid metabolism level in peanut hairy roots

We carried out transcriptome sequencing to better understand how *AhHDA1* alters cell morphology. The metabolism, biomolecular biosynthesis, and photosynthesis pathways were significantly influenced by *AhHDA1*. After adopting stricter criteria, we ranked 98 DEGs that were mostly involved in substance synthesis and energy metabolism. Remarkably, we found that *AhHDA1* overexpression upregulated the expression of most DEGs related to secondary metabolism, and downregulated the expression of most DEGs related to primary metabolism, particularly those related to the photosynthesis pathway. However, most of these DEGs were not affected in *AhHDA1-RNAi* hairy roots (Fig. S2).

The pathways related to secondary metabolism mainly synthesized large numbers of secondary metabolites, such as lignins and flavonoids. Lignin precursor biosynthesis occurs via the phenylpropanoid pathway, and leads to the synthesis of cinnamoyl-CoA esters. CCoAOMTs play an essential role in lignin biosynthesis, and CCoAOMT gene promoters are responsive to signals that control lignin deposition throughout plant development and changes lignin quality in response to environmental conditions (Chen et al., 2000; Zhong et al., 2000). When the flavonoid and isoflavonoid synthesis pathways connect with the phenylpropanoid pathway, CHS and hydroxyisoflavanone synthase are both critical enzymes during this process. Studies on *A. thaliana* showed that in hydroxycinnamoyl-CoA shikimate/quinate hydroxycinnamoyl transferase (HCT)-silenced plants, repression of lignin synthesis and CHS activity lead to the redirection of the metabolic flux into flavonoids (Besseau et al., 2007). Furthermore, the coordinated regulation of genes involved in flavonoid metabolism can redirect flux into the isoflavonoid branch of the phenylpropanoid pathway by reducing the competition for the flavanone substrate. Substance synthesis and energy metabolism (which includes sugar, lipid, and amino acid metabolism) are a significant part of the cell’s primary metabolism pathway.

Our results showed that most substance synthesis and energy metabolism gene (especially photosynthesis gene) expression is downregulated in *AhHDA1*-overexpressed hairy roots, while secondary metabolism pathway gene expression is upregulated (Figs. 8 and 9). This indicated that *AhHDA1* overexpression retards the growth of transgenic hairy roots and may be associated with cell metabolism status.

### The *AhHDA1* mechanism’s influence on metabolic pathways

The results above show the importance of understanding how *AhHDA1* influences metabolic pathways. HDACs erase acetyl groups from histones, resulting in chromatin compaction and gene silencing, and generate acetyl coenzyme A as the product (Ganai et al., 2016). Acetyl coenzyme A can act as a substrate during substance synthesis and in energy metabolism pathways. Therefore, we conjectured that *AhHDA1* could either interact with other proteins to form a protein complex that influences downstream gene expression, or *AhHDA1* mediates substrate activity by controlling acetyl coenzyme A concentration. We considered the first possibility to be the more likely impact factor. To fully understand *AhHDA1*’s influence on downstream genes, it is essential to know which proteins interact with *AhHDA1*.

When screening *Arabidopsis* for RPD3/HDA1 family-interacting proteins, researchers have found that these proteins are involved in plant organogenesis, development, and stress responses. For example, the orchestrated repression of SEP3 by Short Vegetative Phase (SVP), Agamous-Like 24 (AGL24), and Suppressor of Overexpression of Constans 1 (SOC1) is mediated by SAP18, a member of the SIN3 HDAC complex that influences floral patterning (Liu et al., 2009). Additionally, HDA15 has been found to interact directly with PIF3 and NF-YCs in vivo and in vitro. Protein complexes are involved in photosynthesis and photomorphogenesis during the early seedling stage (Liu et al., 2013a; Liu et al., 2013b; Tang et al., 2017). It has been shown previously (Jung et al., 2013) that HDA-interacting proteins are involved in stress responses, and that the cold signaling attenuator High Expression of Osmotically Responsive Gene1 (HOS1) negatively regulates severe responses by interacting with histone deacetylase 6 (HDA6) under short-term cold stress. These examples confirm that RPD3/HDA1 family proteins form complexes with transcriptional factors that are involved in cell development and stress responses.

In this study, we found 37 transcription factors that interact with *AhHDA1* (Table 1). APRR2-like and GLK1-like transcription factors were involved in the ripening process, chlorophyll accumulation, and chloroplast development (Waters, Moylan & Langdale, 2008; Pan et al., 2013; Liu et al., 2016a; Liu et al., 2016b). bHLH77-like, NAC2, and MYB-like transcription factors made up an extensive, functionally, diverse regulatory network that was essential in controlling cell development, metabolism, and stress response. Simultaneously, we detected related transcriptional factor binding sites using promoter analysis, which coincided with the Y2H results (Table S1). These results may provide insight into how *AhHDA1* regulates cell metabolism status.

## Conclusion

In conclusion, our results revealed that overexpressed *AhHDA1* affects apical cell development. Our transcriptome data indicated that this mechanism might also be related to the transcriptional status of metabolism genes. Future studies should focus on identifying the screened *AhHDA1*-interacting proteins, the role of acetyl coenzyme A, and *AhHDA1*’s effect on root development, which would further lay the foundation for improved crop plant varieties.

##  Supplemental Information

10.7717/peerj.10976/supp-1 Supplemental Information 1 Heatmap of substance synthesis and energy metabolism pathway among different hairyroots

10.7717/peerj.10976/supp-2 Supplemental Information 2 Heatmap of photosynthesis pathway among different hairy roots

10.7717/peerj.10976/supp-3 Supplemental Information 3 Transcriptional factor binding sites prediction in six *AhHDA1*-influenced DEGs’ promoters

10.7717/peerj.10976/supp-4 Supplemental Information 4 Primers used in Realtime-PCR and ChIP-PCR test

10.7717/peerj.10976/supp-5 Supplemental Information 5 Sixteen oxidative stress-related DEGs which were influenced by *AhHDA1*

10.7717/peerj.10976/supp-6 Supplemental Information 6 RNASeq processing pipeline

10.7717/peerj.10976/supp-7 Supplemental Information 7 Fig. 1C raw data: root length

10.7717/peerj.10976/supp-8 Supplemental Information 8 Fig. 2A raw data: cell size

10.7717/peerj.10976/supp-9 Supplemental Information 9 Fig. 2B raw data: width to length ratio

10.7717/peerj.10976/supp-10 Supplemental Information 10 Fig. 3D raw data: fluorescence

## Additional Information and Declarations

### Data Availability

The following information was supplied regarding data availability: Data is available at NCBI: PRJNA395475.
