# Supplementary material for: Transcriptome profiling reveals histone deacetylase 1 gene overexpression improves flavonoid, isoflavonoid, and phenylpropanoid metabolism in Arachis hypogaea hairy roots
Source: PeerJ. 2021 Mar 16;9:e10976. doi: 10.7717/peerj.10976 (PMC7977374; doi:10.7717/peerj.10976)
Supplement: Supplemental Information 1 [file peerj-09-10976-s001.pdf]

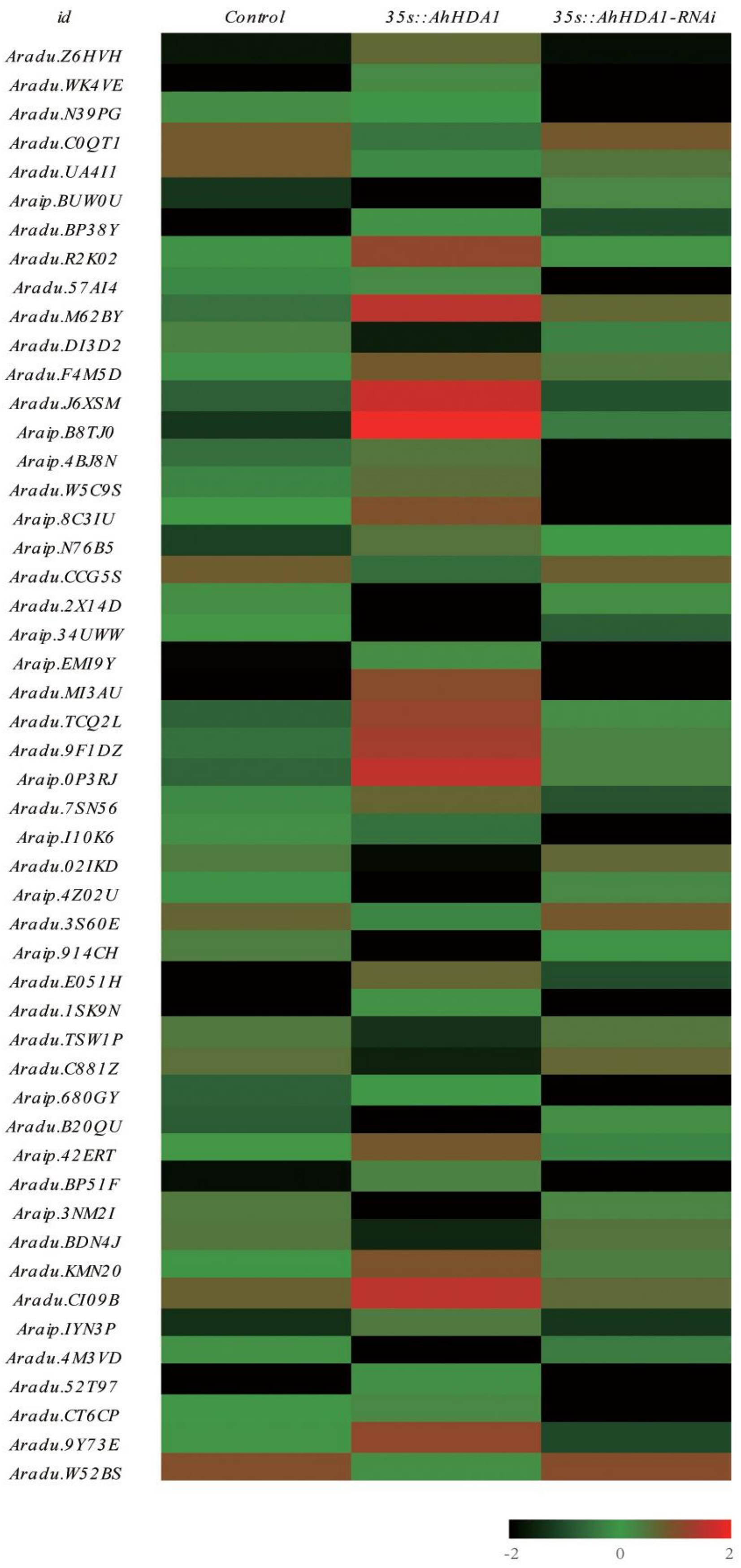

Figure S1 **Heatmap of substance synthesis and energy metabolism pathway among different hairy roots.**
