# Supplementary material for: Transcriptome profiling reveals histone deacetylase 1 gene overexpression improves flavonoid, isoflavonoid, and phenylpropanoid metabolism in Arachis hypogaea hairy roots
Source: PeerJ. 2021 Mar 16;9:e10976. doi: 10.7717/peerj.10976 (PMC7977374; doi:10.7717/peerj.10976)
Supplement: Supplemental Information 6 [file peerj-09-10976-s006.docx]

**1. Library construction and sequencing**

After total RNA was extracted, peanut mRNA was enriched by Oligo(dT) beads. Then the enriched mRNA was fragmented into short fragments using fragmentation buffer and reverse transcripted into cDNA with random primers. Second-strand cDNA were synthesized by DNA polymerase I, RNase H, dNTP and buffer. Then the cDNA fragments were purified with QiaQuick PCR extraction kit, end repaired, poly(A) added, and ligated to Illumina sequencing adapters. The ligation products were size selected by agarose gel electrophoresis, PCR amplified, and sequenced using Illumina HiSeqTM 2500 by Gene Denovo Biotechnology Co. (Guangzhou, China).

**2. Filtering of Clean Reads**

Reads obtained from the sequencing machines includes raw reads containing adapters or low quality bases which will affect the following assembly and analysis. Thus, to get high quality clean reads, reads will be further filtered according to the following rules:
1) removing reads containing adapters;
2) removing reads containing more than 10% of unknown nucleotides (N);
3) removing low quality reads containing more than 50% of low quality (Q-value≤20) bases.
**3. Alignment with Ribosome RNA (rRNA)**

Short reads alignment tool Bowtie2^[1]^ was used for mapping reads to ribosome RNA (rRNA) database. The rRNA mapped reads will be removed. The remaining reads were further used in assembly and analysis of transcriptome.

**4. Alignment with Reference Genome**

The rRNA removed reads of each sample were then mapped to reference genome by TopHat2^[2]^ (version 2.0.3.12), respectively. The alignment parameters were as follows:
1) Maximum read mismatch is 2
2) The distance between mate-pair reads is 50bp
3) The error of distance between mate-pair reads is ±80bp
 After aligned with reference genome, unmapped reads (or mapped very poorly) were then realigned with Bowtie2, the enriched unmapped reads were split into smaller segments which were then used to find potential splice sites. The section and the section position of these short segments were predicted as well. A set of splice sites were built with initial unmapped reads by TopHat2 without relying on the known genes annotation^[3]^.

**5. Transcripts Reconstruction**

The reconstruction of transcripts was carried out with software Cufflinks^[4]^, which together with TopHat2. The program reference annotation based transcripts (RABT) was preferred. Cufflinks constructed faux reads according to reference to make up for the influence of low coverage sequencing. During the last step of assembly, all of the reassembles fragments were aligned with reference genes and then similar fragments were removed. Then we used Cuffmerge to merge transcripts from different replicas of a group into a comprehensive set of transcripts, and then merge the transcripts from multiple groups into a finally comprehensive set of transcripts for further downstream differential expression analysis.

**6. Quantification of Gene Abundance**

Gene abundances were quantified by software RSEM^[5]^. There were two steps for RSEM to quantify gene abundances. Firstly, a set of reference transcript sequences were generated and preprocessed according to known transcripts and new transcripts (in FASTA format) and gene annotation files (in GTF format). Secondly, RNA-seq reads were realigned to the reference transcripts by Bowtie alignment program and the resulting alignments were used to estimate gene abundances. The gene expression level was normalized by using FPKM (Fragments Per Kilobase of transcript per Million mapped reads) method, and the formula is shown as follows:


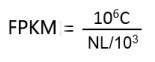


Given FPKM(A) to be the expression of gene A, C to be number of fragments mapped to gene A, N to be total number of fragments that mapped to reference genes, and L to be number of bases on gene A. The FPKM method is able to eliminate the influence of different gene lengths and sequencing data amount on the calculation of gene expression. Therefore, the calculated gene expression can be directly used for comparing the difference of gene expression among samples.
**7. Differentially expressed genes (DEGs) Analysis**

To identify differentially expressed genes across samples or groups, the edgeR package (http://www.rproject.org/) was used. We identified genes with a fold change ≥2 and a false discovery rate (FDR) <0.05 in a comparison as significant DEGs. DEGs were then subjected to enrichment analysis of GO functions and KEGG pathways.
**7.1 GO Enrichment Analysis**

Gene Ontology (GO) is an international standardized gene functional classification system which offers a dynamic-updated controlled vocabulary and a strictly defined concept to comprehensively describe properties of genes and their products in any organism. GO has three ontologies: molecular function, cellular component and biological process. The basic unit of GO is GO-term. Each GO-term belongs to a type of ontology. GO enrichment analysis provides all GO terms that significantly enriched in DEGs comparing to the genome background, and filter the DEGs that correspond to biological functions. Firstly all DEGs were mapped to GO terms in the Gene Ontology database (http://www.geneontology.org/), gene numbers were calculated for every term, significantly enriched GO terms in DEGs comparing to the genome background were defined by hypergeometric test. The calculating formula of P-value is:


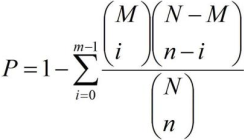


Here N is the number of all genes with GO annotation; n is the number of DEGs in N; M is the number of all genes that are annotated to the certain GO terms; m is the number of DEGs in M. The calculated pvalue were gone through FDR Correction, taking FDR ≤ 0.05 as a threshold. GO terms meeting this condition were defined as significantly enriched GO terms in DEGs. This analysis was able to recognize the main biological functions that DEGs exercise.
**7.2 Pathway Enrichment Analysis**

Genes usually interact with each other to play roles in certain biological functions. Pathway-based analysis helps to further understand genes biological functions. KEGG is the major public pathway-related database^[6]^. Pathway enrichment analysis identified significantly enriched metabolic pathways or signal transduction pathways in DEGs comparing with the whole genome background. The calculating formula is the same as that in GO analysis.


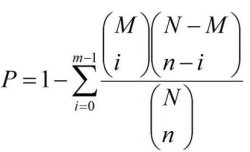


Here N is the number of all genes that with KEGG annotation, n is the number of DEGs in N, M is the number of all genes annotated to specific pathways, and m is number of DEGs in M. The calculated p-value was gone through FDR Correction, taking FDR ≤ 0.05 as a threshold. Pathways meeting this condition were defined as significantly enriched pathways in DEGs.

**Reference**
1. Langmead B, Salzberg S L. Fast gapped-read alignment with Bowtie 2[J]. Nature methods, 2012, 9(4): 357-359.
2. Kim D, Pertea G, Trapnell C, et al. TopHat2: accurate alignment of transcriptomes in the presence of insertions, deletions and gene fusions[J]. Genome biology, 2013, 14(4): R36.
3. Trapnell C, Williams B A, Pertea G, et al. Transcript assembly and quantification by RNA-Seq reveals unannotated transcripts and isoform switching during cell differentiation[J]. Nature biotechnology, 2010, 28(5): 511-515.
4. Trapnell C, Roberts A, Goff L, et al. Differential gene and transcript expression analysis of RNA-seq experiments with TopHat and Cufflinks[J]. Nature protocols, 2012, 7(3): 562-578.
5. Li B, Dewey C N. RSEM: accurate transcript quantification from RNA-Seq data with or without a reference genome[J]. BMC bioinformatics, 2011, 12(1): 1.
6. Kanehisa, M., M. Araki, et al. KEGG for linking genomes to life and the environment. Nucleic Acids Res. 2008.36 (Database issue): D480-4.
